# Quantifying the impact of rising food prices on child mortality in India: a cross-district statistical analysis of the District Level Household Survey

**DOI:** 10.1093/ije/dyx061

**Published:** 2017-04-12

**Authors:** Jasmine Fledderjohann, Sukumar Vellakkal, Zaky Khan, Shah Ebrahim, David Stuckler

First published online: 10 April 2016, *Int J Epidemiol* 2016; 45 (2): 554-564. doi: https://doi.org/10.1093/ije/dyv359

A funding statement was missing for author David Stuckler. The funding statement should have included “D.S. is funded by ERC HRES 313590.”

